# Applications of Latent Growth Mixture Modeling and allied methods to posttraumatic stress response data

**DOI:** 10.3402/ejpt.v6.27515

**Published:** 2015-03-02

**Authors:** Isaac R. Galatzer-Levy

**Affiliations:** Department of Psychiatry, New York University School of Medicine, New York, NY, USA

**Keywords:** Latent growth mixture modeling, latent growth curve analysis, mixture modeling

## Abstract

**Background:**

Scientific research into mental health outcomes following trauma is undergoing a revolution as scientists refocus their efforts to identify underlying dimensions of health and psychopathology. This effort is in stark contrast to the previous focus which was to characterize individuals based on Diagnostic and Statistical Manual of Mental Disorders (DSM) diagnostic status (Insel et al., 2010). A significant unresolved issue underlying this shift is how to characterize clinically relevant populations without reliance on the categorical definitions provided by the DSM. Classifying individuals based on their pattern of stress adaptation over time holds significant promise for capturing inherent inter-individual heterogeneity as responses including chronicity, recovery, delayed onset, and resilience can only be determined longitudinally (Galatzer-Levy & Bryant, 2013) and then characterizing these patterns for future research (Depaoli, Van de Schoot, Van Loey, & Sijbrandij, 2015). Such an approach allows for the identification of phenominologically similar patterns of response to diverse extreme environmental stressors (Bonanno, Kennedy, Galatzer-Levy, Lude, & Elfstom, 2012; Galatzer-Levy & Bonanno, 2012; Galatzer-Levy, Brown, et al., 2013; Galatzer-Levy, Burton, & Bonanno, 2012) including translational animal models of stress adaptation (Galatzer-Levy, Bonanno, Bush, & LeDoux, 2013; Galatzer-Levy, Moscarello, et al., 2014). The empirical identification of heterogeneous stress response patterns can increase the identification of mechanisms (Galatzer-Levy, Steenkamp, et al., 2014), consequences (Galatzer-Levy & Bonanno, 2014), treatment effects (Galatzer-Levy, Ankri, et al., 2013), and prediction (Galatzer-Levy, Karstoft, Statnikov, & Shalev, 2014) of individual differences in response to trauma.

**Method:**

Methodological and theoretical considerations for the application of Latent Growth Mixture Modeling (LGMM) and allied methods such as Latent Class Growth Analysis (LCGA) for the identification of heterogeneous populations defined by their pattern of change over time will be presented (Van De Schoot, 2015). Common pitfalls including non-identification, over identification, and issues related to model specification will be discussed as well as the benefits of applying such methods along with the theoretical grounding of such approaches.

**Conclusions:**

LGMM and allied methods have significant potential for improving the science of stress pathology as well as our understanding of healthy adaptation (resilience).
